# Alternatives surgical training approaches during COVID-19 pandemic

**DOI:** 10.1016/j.amsu.2021.01.057

**Published:** 2021-01-21

**Authors:** Faiz Tuma, Mohamed K. Kamel, Saad Shebrain, Maher Ghanem, John Blebea

**Affiliations:** a General Surgery Department, Central Michigan University College of Medicine, USA; b Genera Surgery Department, Western Michigan University Homer Stryker MD School of Medicine, USA

## Abstract

**Importance:**

Coping with the COIVD-19 global-pandemic major changes in healthcare and educational operational policies, mandates the implementation of alternative surgical curriculum objects (components) to replace some of the traditional face-to-face activities.

**Objective:**

The objective of the study is to review and discuss various alternative curriculum objects (components) that can be used to restructure conventional surgical training curricula during the Declared Healthcare Emergency surgery rotations. The goal is to identify and recommend effective alternative educational activities that are compliant with the new social physical distancing regulations.

**Evidence review:**

Various curricular components and objects were examined. The educational value of the curriculum objects is studied and analyzed in terms of feasibility, knowledge gain/learning effectiveness, the need for facilitation or feedback, and the evaluation. Several curriculum objects were proposed with description of their value and applications.

**Findings:**

The selected and proposed activities include scenario-based MCQ writing exercises, video-based surgical skills interactive training, online learning modules, virtual rounding, reflection assignments, surgical skills simulation training, research education, and medical education learning. Their educational value is described and scaled.

**Conclusion:**

There is urgent and challenging need for surgical training using additional alternative curriculum objects (components). Working with the available resources and experiences is crucial to maximize the learning outcomes. Distance (online) education and educational technology tools and concepts provide a spectrum of valuable educational activities. Further work and studies are needed to optimize their utility.

## Highlights

• The COIVD-19 pandemic has imposed major changes in healthcare and educational operational policies.
• This review discusses alternative curriculum objects that can be used to restructure traditional surgical training curricula.
• Effective alternative educational activities that are compliant with the social distancing regulations were discussed.

## 1 Introduction

On March 11, 2020, the World Health Organization (WHO) declared that the novel Coronavirus (COVID-19) outbreak has reached a global pandemic level [1]. While well-trained, healthcare providers on the frontlines of combating this crisis have since been working tirelessly to provide the optimal medical care for the affected individuals. Medical, surgical, and specialty residency trainees, as well, have been facing significant, unique, and unprecedented challenges. These challenges are not only related to their integral role in providing medical and surgical care to those affected by COVID-19, but also in relation to their responsibility to maintain an appropriate and active learning environment while exercising adequate personal safety measures. Surgical practice changed significantly [2]. In an effort to ensure the safety of healthcare providers and trainees while coping with and providing the best care in anticipation of a patient surge, many healthcare facilities have implemented major strategic shifts in their inpatient and outpatient operational policies [3]. As a result of these challenges, conventional face-to-face educational activities, conferences, and didactics for resident trainees have been interrupted or suspended in the majority of academic institutions and healthcare facilities. In addition, all elective inpatient and outpatient healthcare procedures and services have been suspended, with an increase in the use of tele-health virtual communication. Moreover, social physical distancing practices and limiting gatherings in other educational facilities, such as libraries and simulation labs, have further impacted the learning process for resident trainees. These measures are deemed necessary for the safety and wellbeing of healthcare providers and trainees, but their negative impact on the education and training of the residents should not be overlooked. The efficient use of available resources and training facilitation is of paramount importance. Residents have multiple learning resources, especially with the open educational resources practice and concept, but they need curricular structure and facilitation to maximize their learning experience. Programs are developing their own alternative curricula objects (components) as they navigate this new experience. This article proposes alternative curricular objects (components). The discussion is focused on the educational value with a special emphasis on the structure, objectives, facilitation, and outcomes evaluation.

## 2 Methods

Distance education modalities and technologies have to be assessed scientifically and methodologically as technology forms the backbone of online learning [4]. In order to address the purpose of the study, various curricular components and objects are examined. Their availability, aim, style, purpose suitability, applicability, and effectiveness are studied, analyzed, and summarized. The educational value in terms of fulfilling the training program component is evaluated. The criteria used for evaluation are practicality, effectiveness, need for supervision, and evaluation potential.

1 Feasibility: Usability and feasibility are essential values for online learning activities [5]. In the current circumstances of high demand on multiple activities without preceding sufficient setup preparation, feasibility of the educational activity is important.
2 Gained Knowledge/Objectives: Acquiring knowledge is one of the core competencies of surgery training programs [6,7]. Therefore, the level of knowledge gained, and achievement of rotation objectives are of high importance in any surgical educational activity. For some educators and programs, this might represent the most important aspect.
3 Required supervision: Educational supervision and guidance are among the most important requirements for administration of desirable educational activities [8]. Curriculum objects and educational activities need variable levels of supervision, facilitation, and feedback to ensure objectives are achieved.
4 Evaluation: Assessment of learning and the use of objective means of evaluation continue to impose challenges in surgical training [9]. Surgical curriculum objects and learning activities should have an evaluation component so that learning can be measured, and competency assessed. When a new educational activity results in objective evaluation of residents, it enhances progress through the training milestones.

This assessment structure provides a simple and quick method for surgical educators and programs to choose the activities most suitable for their educational needs.

## 3 Results

After careful and wide review of the common educational technologies, surgical cognitive learning activities, supervision and feedback principles, Accreditation Council for Graduate Medical Education (ACGME) core competencies and milestones, and evaluation requirements, the following curriculum objects (educational activities) are proposed for surgical educators and programs.

1 Writing clinical scenario-based questions;
2 Surgical skills cognitive learning;
3 eLearning modules;
4 Virtual conferencing;
5 Learn from the learner;
6 Review assignments;
7 Surgical skills simulation training;
8 Research education and work; and
9 Teaching and learning.

### 3.1 Writing clinical scenario-based questions

The activity of creating scenario-based questions improves skills in structuring clinical scenarios, choosing a correct solution, reviewing alternatives, and obtaining feedback [10]. Writing questions involves in-depth understanding and review of the clinical situation and identification of available options moving forward through the clinical scenario. Bloom's Taxonomy provides a valuable framework to organize, clarify, and structure educational goals when writing questions [11]. Accordingly, writing questions has the potential to be a highly educational cognitive learning exercise. This activity involves knowledge and skills learning components, and the requirements are minimal. It is extremely feasible, provides fair evaluation of competency, needs minimal to no supervision, and requires some feedback.

### 3.2 Surgical skills cognitive learning

Reviewing surgical procedures among residents under supervision is a valuable and under-utilized training learning exercise [12]. Video training provides flexible opportunities for surgical trainees to learn fundamental technical skills, but it requires high level of supervision [13]. This activity involves running video recordings of selected procedures with a discussion moderated by the attending faculty. It provides cognitive training on observation and analysis of surgical findings, evaluation of the operative status, recognition of unusual findings or complications, planning for management, and evaluation of the application of skills. The only skill component that cannot be covered in this training is the actual hands-on execution of psychomotor skills; this part is best acquired in the simulation (SIM) lab. An additional benefit of this activity is the opportunity to answer resident questions and fill gaps in knowledge.

### 3.3 eLearning modules

There are multiple formats and styles of eLearning modules. Online modules are particularly applicable and practical in the current circumstances. The basic elements of a module are learning materials (source of knowledge), interaction prompts (choices/questions), and evaluation tools. Online learning modules may contain materials from a wide range of learning objects (written information summaries, presentations, books chapters, articles, animation, videos, etc.). Integration of interactivity can be achieved by embedding questions or selections into the modules. A good quality module contains the stated learning outcomes or objectives aligned with teaching activity and assessment [14]. One simple module format is a video-recorded lecture or surgical procedure with integrated, interactive questions, reading suggestions, and/or review of related learning points. One example of an online surgical educational platform is the Surgical Council on Resident Education (SCORE) Portal® which provides residents and residency programs with high-quality educational materials and a structured program for self-learning. SCORE focuses on all areas of general surgery and its related specialties and has been available since 2006. It has been implemented by almost all general surgery residency programs in the US. Additionally, in fall 2019, SCORE merged with the American Board of Surgery (ABS) [6]. Modules demand significant preparation and structuring.

### 3.4 Virtual conferencing

Virtual conferencing to discuss patient conditions was quickly adapted as a replacement for traditional face-to-face rounds. Social physical distancing regulations made this change necessary. This activity demonstrates a highly efficient use of educational technology tools. Via web-conferencing, virtual (distance) conferencing has been in use for several years. Web conferencing has the potential to bring distance learners closer together and enable interactive and collaborative activity that facilitates joint construction of knowledge [15]. The requirements and feasibility of virtual rounds are moderate. Administrative setup and invitations are needed, but many physicians have adapted quickly to the basic technical skills needed to participate. A variety of platforms are conducive to activities of this nature; Microsoft Skype, Zoom, Microsoft Teams, and Cisco WebEx™ are all viable options. Most are user friendly and require minimal technical experience. It is important to choose a platform that maintains patient confidentiality and meets the requirements of the Health Insurance Portability and Accountability Act (HIPPA). Real-time review of patient conditions, assessment and update reports, and imaging can be done efficiently to achieve the objectives of patient care and surgical education. Knowledge or cognitive skills gained depend on the educational quality of the sessions and faculty contribution but sharing knowledge and experience via web conference is comparable to face-to-face sessions. This activity requires high level of supervision and facilitation. Evaluation of trainees’ knowledge and performance during these sessions is efficient and feasible.

### 3.5 Learn with the learner

Learning, as well said and known, is all about sharing. Shared in learning extends beyond exchanging factual information. Searching for specific information, interpreting and connecting facts, conceptualizing principles and applying knowledge are the core elements of the learning process in medical education. Sharing these elements maximizes the educational process and enhances learning skills. DL and ET provides the platform and vehicle for this form of learning. The learning process of a small group of residents studying a particular topic or procedure and exchanging their perception, understanding, analysis. And interpretation of new information and knowledge is recorded. This recorded activity is presented to other learners to enhance and enrich the individual learning environment and outcomes. With DL and OER, sharing such activities between residents, programs or institutions facilitate transfer of knowledge and skills at all times and condictiones. Preparing this activity is moderately demanding, highly educational, and demanding no supervision. But, evaluating resident performance is challenging.

### 3.6 Review assignments

Reviewing topics in a textbook, journal, or similar learning object with the aim of learning pre-determined objectives builds broad knowledge based. The effectiveness of assignment-based instructions has been supported and recommended by the research results over the years [16]. Written assignments to reflect learned objectives is not a new educational activity, but it is not an activity common or familiar to surgical education. This method is more commonly used in academic studies (Diploma, Masters, and Doctorate degrees).

The requirements are high as assignments need to be highly structured and reviewed with effective feedback provided. Evaluation potential is high. “Students can do no better than the assignments they are given.” For effective assignments, educators have to select what to teach and which strategies, methods, and resources which will best guide learning [16].

### 3.7 Surgical skills simulation training

The use of simulation in surgical training is longstanding and continues to evolve significantly. It is becoming an increasingly important educational tool in training surgeons [17]. With an increased need for off-site training in order to minimize contact, simulation can play an even bigger role in training. With the exception of the standardized patient style of simulation, all other modalities of simulation (web-based simulations, virtual reality task trainers, task trainers, virtual patients, high fidelity mannequins) can be effectively used during the current COVID-19 pandemic. The simulation learning environment is stress-free, safe, reproducible, and allows tailoring of training with objective evaluation of performance [18].

Four learning domains (psycho-motor skills, cognitive capabilities, professional affect, and teaching and research skills) can be taught using simulation. Feasibility is variable depending on the available in the institution. The need for supervision depends on the modality of simulation. It is potentially independent training opportunity. Evaluation is also variable depending on the modality, but, in general, it is easily feasible.

### 3.8 Research education and work

The residency training system developed by Stewart Halsted in 1889 includes research as a main principle [19]. Research knowledge, work, and use are essential skills to develop during training. Just as training is essential to gaining proficiency in surgery, competency in the conduct of research also requires training and practice [20]. Three components of research are essential for academic surgeon; understanding the basics of study designs, ability to evaluate a research project/study, and research productivity. Learning research methodology and statistics is becoming easier and more practical with the availability and feasibility of many online courses. These online courses need no extra supervision from the local faculty. Knowledge gain is high if the appropriate courses are selected. Evaluation of learning is simple and reliable.

Many surgical training programs now incorporate designated periods of research activity as part of surgical training [21]. The practice of reviewing, analysing, critically appraising, and potentially applying the results of studies is highly educational. It is feasible and available with minimal arrangements. A good example is virtual journal clubs using web-conferencing. These activities require significant supervision, facilitation and feedback. Evaluating the outcomes is potentially demanding. ‘Research projects are potentially suitable for current circumstances as well. Brainstorming, drafting research proposals, writing and applying for research grants, writing or completing a manuscript, or participating in reviewing or editing manuscripts for journals are examples of valuable scholarly learning activities [12]. Feasibility is variable. High supervision is needed. Evaluation is potentially practical.

### 3.9 Teaching and learning (medical education)

Surgeons as educators is an essential competency for surgical trainees [12]. This competency has been given significant attention by many surgery programs. Medical education is a lifelong learning continuum and skillset that extends from undergraduate to postgraduate and specialization training and beyond [22]. Building surgical education knowledge and skills can be achieved by learning the art and science of education as well as practicing education under supervision. There are numerus resources and learning objects on surgical education. Curriculum objects (components) to teach medical education are easily feasible, result in high and important knowledge gain, may need some supervision, but many may not be easy to evaluate. Practicing teaching by presenting topics accompanied by group feedback under an educators’ supervision is a valuable educational activity that can be done virtually.

## 4 Discussion

The urgent nature and lack of prior preparation and infrastructure made accommodating the ongoing training needs of surgery residents during the pandemic a challenge for many training programs. Working with available resources and experiences is key to replacing many existing face-to-face activities. Surgical training can be viewed as a multifaceted process and structure that aims to result in a good clinician, technician, communicator, scholar, health advocate and professional [21,23]. Highly structured curricula in surgical training have been developed, used, and revised over the years as a result of practice feedback, outcomes assessments, and educational research. In light of the COVID-19 pandemic and its significant impact on healthcare and surgical training, surgical training curricula requires major revision and restructuring to accommodate the various limitations of the pandemic crisis. Shared effort, expertise, and collaboration in order to accommodate the emerging needs of this era are of paramount importance. We believe that this study and its results are a step in that direction. As circumstances evolve, more steps will be needed to evaluate the effectiveness and validity of the new curriculum objects (components).

Teaching is not equal to delivering content. It is the act of designing experiences that facilitate and enable learning [24]. Based on the pre-determined goals and objectives of the training curricula, various educational activities (curriculum objects) can be structured to achieve the desired outcomes of the training. Online education (also called distance education) has been widely utilized for education in many programs and countries [25]. Results from multiple studies have shown that well-designed online medical education activities and courses can facilitate knowledge gains and learning similar to, and at times superior to, traditional face-to-face teaching [26]. Therefore, careful design and structuring of curriculum objects is a key factor in the effectiveness of facilitating knowledge gain and learning. Several web-based training courses have also shown to be effective in enhancing knowledge, confidence, and self-reported practice change outcomes across a variety of clinical subject matter areas [27]. Furthermore, recent studies have compared various online training formats to select and identify the courses that offer the highest-quality educational opportunities in terms of knowledge gain and participant satisfaction and gains [28]. There are many application guidelines and reviews on the concept and application of online learning. Literature on content design for training purposes has also been published [[29], [30], [31]]. Our selection of components was based on achieving the objectives in the current circumstances using common and available resources. The evaluation and recommendation of the selected activities is designed to provide baseline alternatives to traditional curriculum. Further critical review of experiences in additional programs supported by outcomes assessment will improve our knowledge and further develop understanding of effective modalities.

## 5 Conclusions

The lack of prior preparation for such urgent needs made it more challenging for training programs to accommodate the ongoing training needs of surgery residents. Working with the available resources and experiences is key to replace many sustained face-to-face activities. Educational technology offers tremendous support to current training efforts via highly efficient communication, materials storage and sharing, editing and producing capabilities, and the convenience of asynchrony.

Our review and recommendations are merely a foundation that can be further developed, modified, or replaced according to the needs of each program. Therefore, continuous re-evaluation, feedback, and collaborative efforts to update the available curriculum objects will be essential to developing higher quality surgical training.

Studies have also supported the use of distance (online) education in training medical students [32,33], especially in resource-limited settings. Further work and studies are needed to provide recommendations of curriculum objects for medical students. In the current COVID-19 pandemic, these studies should be given priority.

## Ethical approval

This is a review article. Therefore, ethical committee approval was not required.

## Author contribution

Study concept or design: Faiz Tuma, Mohamed K. Kamel, Saad Shebrain, Maher Ghanem, John Bleba.

Data collection: Faiz Tuma, Mohamed K. Kamel, Saad Shebrain, Maher Ghanem, John Bleba.

Data interpretation: Faiz Tuma, Mohamed K. Kamel, Saad Shebrain, Maher Ghanem, John Bleba.

Writing the paper: Faiz Tuma, Mohamed K. Kamel, Saad Shebrain, Maher Ghanem, John Bleba.

## Consent

Not applicable.

## Registration of research studies

This is a review article, and therefore is not a research that involve human subjects.

## Guarantor

Faiz Tuma, MD.

## Funding

None.

## Provenance and peer review

Not commissioned, externally peer reviewed.

## Declaration of competing interest

None.

## References

[1] WHO Director-General's Opening Remarks at the COVID19 Media Briefing in March 2020.

[2] O'Logbon J. (2020). What can surgery learn from other high-performance disciplines?. Ann Med Surg (Lond)..

[3] Michail Sideris, Hanrahan John Gerrard, Papalois Vassilios (2020). COVID-19 and surgical education: every cloud has a silver lining. Annals of Medicine and Surgery.

[4] Bates A.W., Poole Gary (2003). Effective Teaching with Technology in Higher Education.

[5] Wang H.Y., Liu T.C., Chou C.Y., Liang J.K., Chan T.W., Yang S. (2004). A framework of three learning activity levels for enhancing the usability and feasibility of wireless learning environments. J. Educ. Comput. Res..

[6] ABS. SCORE (2019). Surgical Council on resident education. http://www.absurgery.org/?aboutscre.

[7] ACGME, ABS (2015). The general surgery milestone project. http://www.acgme.org/portals/0/pdfs/milestones/surgerymilestones.pdf.

[8] Jahanian R., Ebrahimi M. (2013). Principles for educational supervision and guidance. J. Socio Res..

[9] Peracchia A. (2001). Presidential Address: surgical education in the third millennium. Ann. Surg..

[10] Brame C. (2013). Writing Good Multiple Choice Test Questions. https://cft.vanderbilt.edu/guides-sub-pages/writing-good-multiple-choice-test-questions/.

[11] Bloom B.S. (1956). Taxonomy of Educational Objectives. The Classification of Educational Goals. Handbook 1: Cognitive Domain.

[12] Faiz T., Kamel M.K., Shebrain S., Ghanem M., Blebea J. (2020 Dec 18). Options of surgical curriculum structure and objects during COVID-19 pandemic. Surg. Innovat..

[13] Jowett N., LeBlanc V., Xeroulis G., MacRae H., Dubrowski A. (2007). Surgical skill acquisition with self-directed practice using computer-based video training. Am. J. Surg..

[14] Burge A. (2020). How to Design Effective Teaching Modules. http://www.absurgery.org/default.jsp?aboutscre.

[15] Siemens G. (2004). Connectivism: a learning theory for a digital age. http://www.itdl.org/journal/jan_05/article01.htm.

[16] Dougherty E. (2012). Assignments matter. ASCD learn teach lead. http://www.ascd.org/publications/books/112048/chapters/Why-Assignments-Matter.aspx.

[17] Trehan K., Kemp C.D., Yang S.C. (2014). Simulation in cardiothoracic surgical training: where do we stand?. J. Thorac. Cardiovasc. Surg..

[18] Hoznek A., Salomon L., de la Taille A. (2006). Simulation training in video-assisted urologic surgery. Curr. Urol. Rep..

[19] Sealy W.C. (1999). Halsted is dead: time for change in graduate surgical education. Curr. Surg..

[20] Omigbodun A.O. (2012). The intersection of research and surgical training. J West Afr Coll Surg.

[21] Javed M.S., Harrison E., Taylor I. (2003). The role of research in surgical training. Bull. Roy. Coll. Surg. Engl..

[22] Bashook P.G., Parboosingh J. (1998). Recertification and the maintenance of competence. BMJ.

[23] Agha R.A., Fowler A.J., Sevdalis N. (2015). The role of non-technical skills in surgery. Ann Med Surg (Lond).

[24] Qiao Y.Q., Shen J., Liang X. (2014). Using cognitive theory to facilitate medical education. BMC Med. Educ..

[25] Mayadas AF, Bourne J, Bacsich P. Online education today. Science. 009;323(5910):85–89. doi:10.1126/science.1168874. 10.1126/science.1168874 19119225

[26] Fordis M., King J.E., Ballantyne C.M. (2005). Comparison of the instructional efficacy of Internet-based CME with live interactive CME workshops: a randomized controlled trial. J. Am. Med. Assoc..

[27] Curran V., Lockyer J., Sargeant J., Fleet L. (2006). Evaluation of learning outcomes in web-based continuing medical education. Acad. Med..

[28] Curran V.R., Fleet L.J., Kirby F. (2010). A comparative evaluation of the effect of internet-based CME delivery format on satisfaction knowledge and confidence. BMC Med. Educ..

[29] Ellaway R, Masters K. AMEE Guide 32: e-learning in medical education part 1: learning, teaching, and assessment. Med. Teach., 30:455–473. doi: 10.1080/01421590802108331. 10.1080/01421590802108331 18576185

[30] Cook D.A., Dupras D.M. (2004). A practical guide to developing effective web-based learning. J. Gen. Intern. Med..

[31] Sisson S.D., Hill-Briggs F., Levine D. (2010). How to improve medical education website design. BMC Med. Educ..

[32] Cirigliano Matthew M., Guthrie Charles D., Pusic Martin V. (2020). Click-level learning analytics in an online medical education learning platform. Teach. Learn. Med..

[33] Kelly Gillingham, Eggleton Kyle, Goodyear-Smith Felicity (2020). Is reflective learning visible in online discussion forums for medical students on general practice placements? A qualitative study. Teach. Learn. Med..

